# Dosimetric dependence of the dimensional characteristics on a lead shield in electron radiotherapy: a Monte Carlo study

**DOI:** 10.1120/jacmp.v10i2.2918

**Published:** 2009-04-29

**Authors:** James C L Chow, Grigor N Grigorov

**Affiliations:** ^1^ Radiation Medicine Program, Princess Margaret Hospital and Department of Radiation Oncology University of Toronto Toronto Ontario Canada; ^2^ Department of Physics University of Waterloo Waterloo Ontario Canada; ^3^ Department of Physics Ryerson University Toronto Ontario Canada; ^4^ Medical Physics Department Grand River Regional Cancer Center Kitchener Ontario Canada

**Keywords:** electron radiotherapy, Monte Carlo, Pb shielding, electron dosimetry

## Abstract

This study investigates the dosimetric dependence of the dimension of a lead (Pb) layer for shielding using clinical electron beams with different energies. Monte Carlo simulations were used to generate phase space files for the 4, 9 and 16 MeV electron beams produced by a Varian 21 EX linear accelerator using the EGSnrc‐based BEAMnrc code, and validated by measurements using films. Pb layers with different thicknesses (2, 4, 6 and 8 mm) and diameters (2.5, 3, 3.5 and 4 cm) were placed at the center of an electron field on a solid water phantom. Beam profiles were determined at the depth of maximum dose $\left(d_{m}\right)$ using Monte Carlo simulations. The dose profiles under the Pb layer at $d_{m}$, including the penumbra at the edge of the layer and relative dose at the central beam axis (CAX), were studied with varying thicknesses and diameters of Pb. It is found that 2 mm of Pb is adequate to provide 5 half value layer (HVL) attenuation for the 4 MeV electron beams, and the beam profiles at $d_{m}$ are dependent on the diameter but not the thickness of the Pb. However, for the 9 and 16 MeV electron beams, the relative dose at the CAX and $d_{m}$ depends on both the thickness and diameter of the Pb layer. For 8 mm thickness of Pb, 4 and 5 HVL attenuation of electron beams with energies of 9 and 16 MeV can be achieved at $d_{m}$, respectively. Moreover, the beam profile under the Pb layer at $d_{m}$ depends on: (1) the penumbra region at the edge of the Pb layer; (2) the beam attenuation varying with the thickness of the Pb layer; (3) the electron side scatter contributing to the CAX under the Pb layer; and (4) the photon contamination produced by the Pb layer. A parameter called “shielding area factor” (defined as the ratio of the length between two points of 50% relative doses in the beam profile at $d_{m}$ to the diameter of the Pb layer) is suggested to predict the required size and thickness of Pb for shielding a target with known dimension at $d_{m}$. The dosimetric data calculated by Monte Carlo simulations in this study are useful to select the suitable thickness and size of Pb for the protection of critical tissue in electron radiotherapy.

PACS number: 87.53.Bn; 87.55.kh and 87.55.km.

## I. INTRODUCTION

In electron radiotherapy, superficial lesions on the eye or eyelid can be treated by low‐energy electron beams (4 – 9 MeV). To protect critical structures such as the lens during treatment, lead (Pb) is used as shielding.^(1)^ A patient‐specific electron cutout inserted in the bottom of an electron applicator is used together with a layer of Pb (or commercially available eye shield made of Pb or tungsten (W)) placed on the patient surface.^(2)^ ^,^ ^(3)^ In this treatment setup and geometry, there are several dosimetric concerns about the dose distribution at the prescription depth or depth of maximum dose $\left(d_{m}\right)$. One of the common concerns is the dose enhancement to the critical tissue due to the electron backscatter from the Pb shield.^(1)^ ^,^ ^(4)^ ^,^ ^(5)^ There have been many studies regarding Monte Carlo simulations or measurements using TLD, films, metal‐oxide‐semiconductor field effect transistor (MOSFET), and plane parallel ionization chamber on the electron backscatter from Pb irradiated by photon and electron beams.^(^ ^1^ ^,^ ^4^ ^,^ ^5^ ^–^ ^11^ ^)^ Other studies of electron backscatter focused on commercial eye shields made of either Pb or W.^(2)^ ^,^ ^(3)^ A more general approach using different thicknesses of Pb layers was used in this study to determine the dosimetry of backscatter at the Pb‐tissue interface.

Results of Monte Carlo simulations studying the dependences of the electron backscatter on the beam obliquity, energy, thickness of Pb, and depth of the Pb‐tissue interface using the EGSnrc‐based code have recently been published.^(11)^ In addition, the relatively new MOSFET dosimeter was examined to determine the electron‐backscattered dose accurately at the Pb‐tissue interface.^(12)^ ^,^ ^(13)^ However, related studies concerning the effect of the dimensions of the Pb layer on the dose distribution under the Pb shield (at the prescription depth or $d_{m}$) is lacking. The dosimetry near the $d_{m}$ and build‐up region for the low‐energy electron beam (i.e. 4 MeV) is more complex compared to higher energies due to the larger angular scattering cross‐section and shorter effective range.^(14)^ ^,^ ^(15)^ In addition, when the Pb layer is placed on the patient/phantom surface under the electron applicator with cutout, the dosimetry in the penumbra regions and central beam axis (CAX) of the beam profile depends on the dimensional characteristics of the Pb layer, such as its diameter and thickness. The aim of this study is to determine the dosimetric characteristics such as the beam profile, relative dose at the CAX and penumbra width at $d_{m}$ with varying diameters and thicknesses of the Pb layer and different electron beam energies.

To determine this new dosimetric data for the Pb shield, Monte Carlo simulations, validated by measurements, were used to calculate the beam profiles at $d_{m}$ for different Pb shields and electron beam energies. The advantages of using Monte Carlo simulations instead of measurements to determine the dose distributions are: (1) minimizing the time and effort needed in measuring the beam profiles with a scanning water tank since only measurements for validation are needed; (2) avoiding human error in the experimental setup and fabrications of cutouts and Pb layers in the measurement; and (3) simulation results account for the electron scattering for the low‐energy electron beam and electron disequilibrium effect for the high‐energy electron beam.^(16)^

In this study, electron beams with energies of 4, 9 and 16 MeV produced by a Varian 21 EX linear accelerator (Linac) were used. Although low‐energy electron beams were used to treat superficial lesions, dosimetric data of the 16 MeV electron beams were calculated here for a more thorough analysis and comparison. Kodak XV radiographic film was used to validate Monte Carlo simulations.

## II. MATERIALS AND METHODS

### A. Experimental Setup

**Figure 1:**
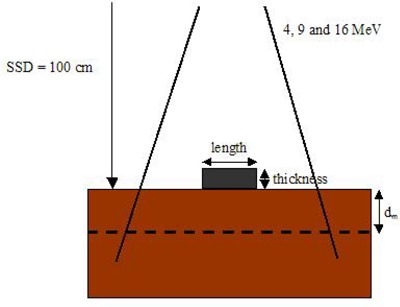
Schematic diagram showing the experimental setup for Monte Carlo simulations to calculate the beam profiles at $d_{m}$ (broken line) for the 4, 9 and 16 MeV electron beams. The SSD was equal to 100 cm. The diameters (lengths) and thicknesses of Pb layers were varied from 2.5 – 4 cm and 2 – 8 mm, respectively.

The experimental setup and geometry for studying the dosimetry at the prescription depth or $d_{m}$ can be seen in Fig. 1. Pb layers with thicknesses ranging from 2 – 8 mm were placed on a solid water slab $\left(30\times 30\;{\text{cm}}^{2}\right)$ with source‐to‐surface distance (SSD)=100 cm. Four sizes of Pb layers with diameters equal to 2.5, 3, 3.5 and 4 cm were used on the phantom surface. The 4, 9 and 16 MeV electron beams produced by the Varian 21 EX Linac with $10\times 10\;{\text{cm}}^{2}$ applicator and circular cutout (diameter=10 cm) were used in the measurement. Beam profiles at $d_{m}$ (i.e. 0.7 cm for 4 MeV, 2.2 cm for 9 MeV, and 3 cm for 16 MeV) were calculated using Monte Carlo simulations (EGSnrc–based code) after validations by film measurements.

### B. Monte Carlo Simulation

The beam profiles for the experimental geometry and setup, as shown in Fig. 1, were calculated using the EGSnrc Monte Carlo code.^(^ ^17^ ^,^ ^18^ ^)^ The BEAMnrc^(19)^ ^,^ ^(20)^ and DOSXYZnrc^(21)^ code were used to calculate the phase space file for the electron beams and dose in the phantom, respectively.

#### B.1. Phase Space File for the Electron Beam

Phase space files of the types, positions, energies, and directions of particles for the electron beams with energies of 4, 9 and 16 MeV were generated using the BEAMnrc code (version 4‐r2‐2‐4). The $10\times 10\;{\text{cm}}^{2}$ applicator and circular cutout were used in the phase space file. The initial mean energy $\left(E_{o}\right)$ of the electron beam with SSD=100 cm produced by the Linac was estimated using the measured electron range at 50% of depth dose $\left(R_{50}\right)$ in the commissioning. The following equation relating $E_{0}$ and $R_{50}$ was used:^(22)^

(1) $$E_{o}=C_{4}\times R_{50}$$

Original recommendations by the AAPM TG‐21 protocol suggested a value for $C_{4}$ of 2.33 Mev/cm for water.^(23)^ However, in the clinical energy range of 1 – 50 MeV, Monte Carlo calculations using EGS4 show that $C_{4}=2.4\;\text{MeV}/\text{cm}$.^(24)^ ^,^ ^(25)^ The primary electron energy was estimated by comparing the percentage depth dose (PDD) calculated by the assumed monoenergetic energy of the primary electron using Monte Carlo simulation and the measurement. In this study, the primary electron energies for the 4, 9 and 16 MeV beams were estimated to be 4.17, 10.40 and 17.51 MeV, respectively, with deviation of the PDD between the Monte Carlo simulation and measurement <±2%, after iterative readjustment of the primary electron energies.^(16)^ ^,^ ^(26)^

In our Monte Carlo simulation, the phase space file (containing at least 10 million particles) was not recycled more than five times when running DOSXYZnrc.^(21)^ The recycling of a particle does not introduce any bias, as long as the statistical weight and the variance are accurately calculated. The Parameter Reduced Electron Step Transport Algorithm II (PRESTA II) was used as the electron‐step algorithm with the user‐adjustable parameters set at their default values.^(27)^ ^,^ ^(28)^ The parameters of the electron cutoff energy (ECUT) and maximum fractional energy loss per electron step (ESTEPE) were set to 700 keV and 0.25, respectively.^(11)^

#### B.2. Dose Calculation With the Pb Layer

The beam profiles in the in‐plane or x‐axis according to Fig. 1 were calculated using the EGSnrc–based DOSXYZnrc code. The solid water phantom and beam setup in Fig. 1 were input to the DOSXYZnrc with voxel volume equal to $0.2\times 0.2\times 0.1\;{\text{cm}}^{3}$ for the x, y and z direction, respectively. For electron beams with energies of 4, 9 and 16 MeV, different diameters (2.5, 3, 3.5 and 4 cm) and thicknesses (2, 4, 6 and 8 mm) of Pb layers were placed at the center of a circular field with diameter equal to 10 cm. The space between the applicator and the phantom surface was 5 cm and the SSD (phantom surface) was set to 100 cm. Each simulation was finished using 50 million histories. Under this calculation tally, the relative statistical dose error (that is the uncertainty as a fraction of dose in the voxel) was around ±1% for all electron beam energies.^(21)^ The parameter of ECUT for DOSXYZnrc was set to 521 keV. The computing time was about 0.5 – 1 hour using a single AMD Athlon 64 X2 4200+processor with 1 GB RAM.

### C. Measurement for Validation of Monte Carlo Simulation

Dose measurements were carried out using Kodak XV films with experimental setup in Fig. 1 to determine the beam profiles at $d_{m}$. The film was calibrated with electron beam energies (4, 9 and 16 MeV) produced by the Linac using the standard $10\times 10\;{\text{cm}}^{2}$ applicator and cutout. The absolute dose of 1 cGy per monitor unit (MU) for the electron beam was calibrated by a plane parallel ionization chamber (PTW 23343 Markus) placed at the depth of reference $\left(d_{\text{ref}}\right)$ in a water tank at SSD=100 cm using the AAPM TG‐51 protocol.^(29)^ The absolute dose point was then cross‐calibrated with a Farmer‐type ionization chamber (CAPINTEC PR‐06C) and solid water phantom for the same beam and setup geometry. The solid water slab (CNMC, Tennessee, USA) is designed for photon and electron beam calibration with its mass and volume electron densities, electron and photon transmission characteristics, and with physical dimensions virtually the same as those in liquid water.

For the film calibration, films taken from the same batch were placed in the solid water phantom at $d_{\text{ref}}$ (SSD=100 cm). Different MUs, in the film linear dose sensitivity range (0 – 1 Gy), were used to expose the film for the 4, 9 and 16 MeV electron beams. The exposed films were developed in a Kodak X‐Omat 2000 film processor and analyzed using the Vidar VX‐16 densitometer with RIT 113 radiation therapy dosimetry software, version 4 (Radiological Imaging Technology, Inc., Colorado Springs, CO, U.S.A.). The sensitometric curve fitting was done by the piecewise polynomial routine in the software. The uncertainty of the film dosimetry is within ±3%.^(30)^ The measured results using films were used to validate Monte Carlo simulations.

## III. RESULTS

**Figure 2:**
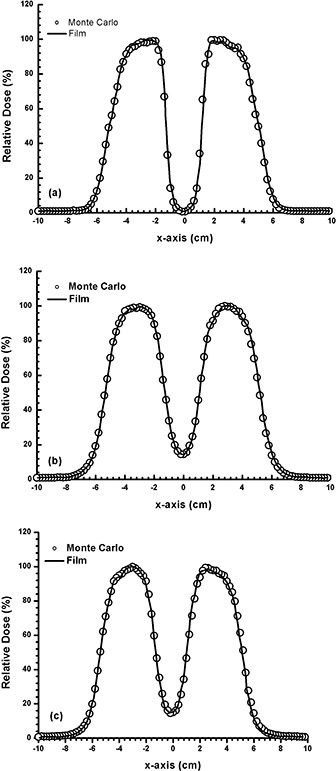
Beam profiles measured and calculated using films and Monte Carlo simulations for: (a) 4 MeV electron beams with 2 mm thickness of Pb at depth=0.7 cm, (b) 9 MeV electron beams with 4 mm thickness of Pb at depth=2.2 cm, and (c) 16 MeV electron beams with 6 mm thickness of Pb at depth=3 cm. The diameter of the Pb layer was equal to 2.5 cm and experimental setup as in was used.

Figure 2 shows the beam profiles at $d_{m}$ for both measured and calculated data using films and Monte Carlo simulations as outlined in the experimental setup in Fig. 1. The diameter of the Pb layer was equal to 2.5 cm and the thickness of the layer was equal to 2, 4 and 6 mm for the electron beams with energies of 4 MeV (Fig. 2(a), 9 MeV (Fig. 2(b) and 16 MeV (Fig. 2(c), respectively. All beam profiles in Fig. 2 were normalized to the maximum doses. It can be seen that the doses calculated by Monte Carlo simulations agree well with the measurements, and therefore the Monte Carlo model for the Linac was verified.

**Figure 3:**
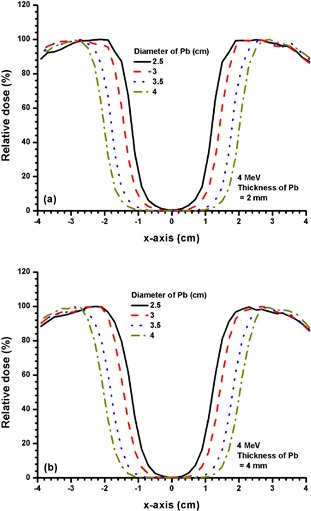
Beam profiles for Pb layers with diameters of 2.5, 3, 3.5 and 4 cm and thicknesses of (a) 2 mm, and (b) 4 mm. The 4 MeV electron beams with the $10\times 10\;{\text{cm}}^{2}$ applicator and circular cutout were used. It can be seen that both 3(a) and 3(b) had relative doses at the CAX equal to zero.

**Figure 4:**
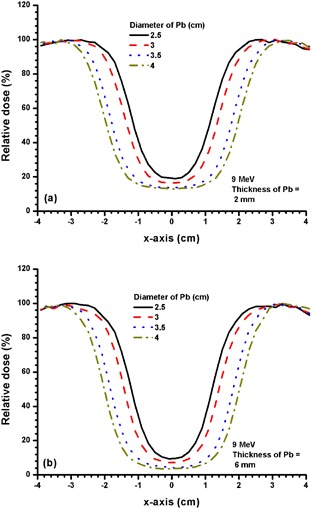
Beam profiles for Pb layers with diameters of 2.5, 3, 3.5 and 4 cm and thicknesses of (a) 2 mm, and (b) 6 mm. The 9 MeV electron beams with the $10\times 10\;{\text{cm}}^{2}$ applicator and circular cutout were used. The relative dose at CAX in 4(a) decreased from 19.5% to 13.0% when diameter of the Pb layer increased from 2.5 to 4 cm. In 4(b), the relative dose at the CAX decreased from 9.13% to 3.4% when the diameter of the Pb layer increased from 2.5 to 4 cm.

**Figure 5:**
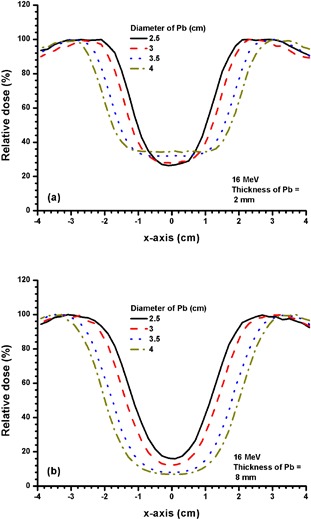
Beam profiles for Pb layers with diameters of 2.5, 3, 3.5 and 4 cm and thicknesses of (a) 2 mm, and (b) 8 mm. The 16 MeV electron beams with the $10\times 10\;{\text{cm}}^{2}$ applicator and circular cutout were used. The relative dose at the CAX for 5(a) increased from 26.7% to 34.5% when the diameter of the Pb layer increased from 2.5 to 4 cm. The relative dose at the CAX for 5(b) decreased from 15.8% to 6.9% with the same increase of diameter of the Pb layer as in 5(a).

Figures 3(a) and 3(b) show the beam profiles at $d_{m}$ for 2 and 4 mm thickness of Pb with different diameters (2.5, 3, 3.5 and 4 cm) using the 4 MeV electron beam. Figures 4 and 5 show beam profiles with similar experimental setup as in Fig. 3, with electron beam energies of 9 MeV and 16 MeV and thicknesses of 6 and 8 mm of Pb. Thicker Pb layers used in Figs. 4 and 5 were due to the increase of electron beam energy.

**Figure 6:**
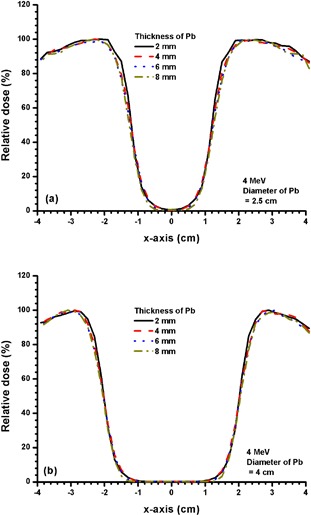
Beam profiles for Pb layers with diameters of (a) 2.5 and (b) 4 cm and thicknesses of 2, 4, 6 and 8 mm. The 4 MeV electron beams with the $10\times 10\;{\text{cm}}^{2}$ applicator and circular cutout were used. The relative doses at the CAX of all beam profiles for 6(a) and 6(b) were equal to zero.

**Figure 7:**
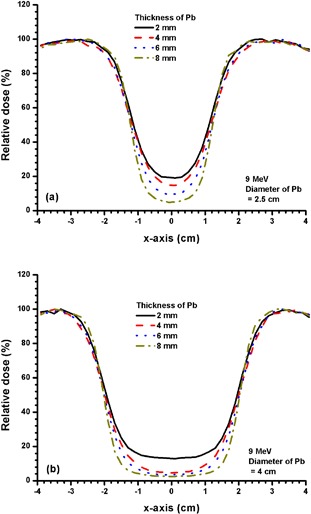
Beam profiles for Pb layers with diameters of (a) 2.5 and (b) 4 cm and thicknesses of 2, 4, 6 and 8 mm. The 9 MeV electron beams with the $10\times 10\;{\text{cm}}^{2}$ applicator and circular cutout were used. In 7(a), the relative dose at the CAX decreased from 19.3% to 4.8% when the thickness of the Pb layer increased from 2 to 8 mm. When the diameter of the Pb layer is increased to 4 cm (7(b)), the relative dose at the CAX decreased from 13.2% to 3.5% with thickness of the Pb layer increased from 2 to 8 mm.

**Figure 8:**
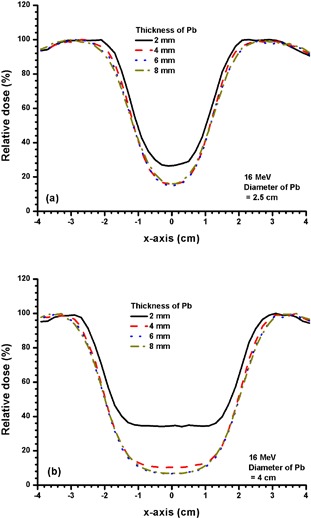
Beam profiles for Pb layers with diameters of (a) 2.5 and (b) 4 cm and thicknesses of 2, 4, 6 and 8 mm. The 16 MeV electron beams with the $10\times 10\;{\text{cm}}^{2}$ applicator and circular cutout were used. When the thickness of the Pb layer increased from 2 to 8 mm, the relative dose at the CAX decreased from 26.9% to 14.9% for 8(a), and from 34.3% to 6.6% for 8(b).

Figures 6(a) and 6(b) show beam profiles for Pb layers with diameters of 2.5 and 4 cm using the 4 MeV electron beam. The thicknesses of Pb used were 2, 4, 6 and 8 mm. Figures 7 and 8 show beam profiles for Pb layers similar to Fig. 6, except the electron beam energy was changed to 9 MeV and 16 MeV. The statistical uncertainties for beam profiles in Figs. 3 – 8 calculated using Monte Carlo simulations are within ±1%, as discussed in Section II.B.2 above.

**Figure 9:**
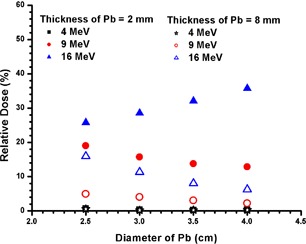
Relative dose at the CAX vs. diameter of the Pb layer. The 4, 9 and 16 MeV electron beams were used with thickness of Pb equal to 2 and 8 mm. It should be noted that data points for the 4 MeV electron beams with Pb thicknesses of 2 and 8 mm were overlapped in the figure.

**Figure 10:**
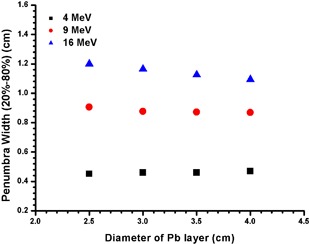
Penumbra width (20%‐80%) at the edge of the Pb layer vs. diameter of the Pb layer for the 4, 9 and 16 MeV electron beams. The beam profiles were measured at 0.7 cm (4 MeV), 2.2 cm (9 MeV) and 3 cm (16 MeV), respectively. The thickness of the Pb layer was equal to 2 mm.

Figure 9 shows the relative dose at the CAX for the 2 and 8 mm thicknesses of the Pb using the 4, 9 and 16 MeV electron beams, where the diameter of the Pb layer was equal to 2.5 cm. Figure 10 shows the penumbra width (20% – 80%) under the edge of the Pb layer vs. its diameter at $d_{m}$ for the 4, 9 and 16 MeV electron beams. In Fig. 10, the thickness of Pb layers for the 4, 9 and 16 MeV electron beams was 2 mm.

A parameter called the shielding area factor, reflecting the shielding area efficiency of the Pb layer at $d_{m}$, was defined in this study. The shielding area factor is defined as:

(2) $$Shielding\;area\;factor=\frac{l(d_{m},D_{50\%})}{Diameter\;of\;Pb\;shield}$$

where 1 is the length between two points of 50% relative dose $\left(D_{50\%}\right)$ in the beam profile at $d_{m}$. The ratio of 1 to the physical length (diameter) of the Pb layer/shield on the patient/phantom surface gives the shielding area factor. The changes in shielding area factor with different thicknesses and diameters of Pb layers for the 4 MeV (5 half value layer (HVL) attenuation), 9 MeV (3 and 4 HVL attenuation), and 16 MeV (3 HVL attenuation) electron beams are shown in Tables 1(a), 1(b), 1(c), and 1(d), respectively. The diameter of the Pb layer was equal to 2.5 cm in the table.

**Table 1(a):** 4 MeV (5HVL)

	*Diameter of Pb shield (cm)*			
Thickness of Pb	2.5	3.0	3.5	4.0
2 mm	0.39	0.47	0.62	0.67
4 mm	0.43	0.49	0.64	0.68
6 mm	0.47	0.52	0.67	0.70
8 mm	0.52	0.59	0.73	0.76

**Table 1(b):** 9 MeV (4HVL)

	*Diameter of Pb shield (cm)*			
Thickness of Pb	2.5	3.0	3.5	4.0
6 mm	NA	NA	0.39	0.46
8 mm	0.24	0.41	0.60	0.66

**Table 1(c):** 9 MeV (3HVL)

	*Diameter of Pb shield (cm)*			
Thickness of Pb	2.5	3.0	3.5	4.0
4 mm	NA	0.27	0.56	0.60
6 mm	0.35	0.48	0.66	0.69
8 mm	0.61	0.64	0.79	0.79

Shielding area factors with different thicknesses of Pb for the (a) 4 MeV electron beams with 5 HVL attenuation, (b) 9 MeV electron beams with 4 HVL attenuation, (c) 9 MeV electron beams with 3 HVL attenuation, and (d) 16 MeV electron beams with 3 HVL attenuation. The factors were measured at dm of 0.7, 2.2 and 3 cm for the 4, 9 and 16 MeV electron beams, respectively. The diameter of the Pb layer was equal to 2.5 cm. The percentages on top of each sub‐table represent the attenuation result.

## IV. DISCUSSION

### A. Effect of the Area of the Pb Layer on the Beam Profile

Beam profiles for 2 and 4 mm thickness of Pb with different diameters at $d_{m}$ can be seen in Figs. 3(a) and 3(b) for the 4 MeV electron beams. It is obvious that both have zero relative doses at the CAX and similar penumbras. Moreover, a 2 mm thickness of Pb is found to be adequate to provide shielding at the CAX. The relative doses at the CAX in Fig. 3 are less than 3% (5 HVL). However, the Pb layer cannot provide the same area of shielding at $d_{m}$ compared to its physical size because of the finite penumbra width at the edge of the layer.

When the electron beam energy is increased to 9 MeV (Fig. 4), the shielding efficiency is decreased as reflected by the relative dose at the CAX. For example, increasing the thickness from 2 to 6 mm for a 2.5 cm diameter Pb layer can decrease the relative dose at the CAX from 19.3% to 9.6% (Fig. 4), but it is impossible for the Pb layer to achieve 5 HVL attenuation (Fig. 3). However, it is seen in Fig. 4 that the relative doses at the CAX depend on the diameter of the Pb layer. The larger the Pb layer (from 2.5 to 4 cm diameter with 6 mm thickness) used on the phantom surface, the lower the minimum relative dose (from 9.6% to 3.5% in the beam profile). This is due to the movement of the penumbra at the edge of the Pb layer towards the CAX when the diameter of the layer decreases. Therefore, when the shielding area is decreased, the shielding efficiency for the dose is also decreased because of the penumbra width of the Pb layer. This penumbra width depends on the electron beam energy.

When the electron beam energy is increased to 16 MeV, a 2 mm thickness of Pb can only attenuate the beam to about 30% – 35% at the CAX, depending on the size of the layer (Fig. 5(a). However, with an 8 mm thickness of Pb reaching the practical shielding limit, the relative dose at the CAX can only be attenuated to about 10% – 15%, which is about 3 HVL at the CAX (Fig. 5(b). In Fig. 5(a), it is interesting to note that the minimums of the relative dose distribution for diameters of Pb equal to 3.5 and 4 cm extend to both sides of the CAX. This is because of the narrower penumbra width for the high electron beam energy due to a lower scattering cross‐section. Therefore, when the shielding area is increased to 3.5 and 4 cm diameter, the two penumbra regions are moved away from the CAX (Fig. 5(a), resulting in a minimum relative dose region instead of a point. It should be noted that such phenomenon would not happen for thicker Pb layers (Fig. 5(b) due to a higher attenuation of Pb for the electron beams.

### B. Effect of the Thickness of the Pb Layer on the Beam Profile

The dose distribution at $d_{m}$ does not change significantly with variations in the thickness and diameter of the Pb layer for electron beams with a low energy of 4 MeV. This can be seen in Figs. 6 (a) and (b). All beam profiles do not change significantly when the small (2.5 cm diameter, Fig. 6(a) and larger (4 cm diameter, Fig. 6(b) Pb layers were used with different thicknesses. The profiles show that for low‐energy electron beams, the diameter and thickness of the Pb layer would not affect the dose distribution at $d_{m}$. In addition, a 2 mm thickness of Pb is seen to be adequate for shielding (Fig. 6).

However, for the higher electron beam energy of 9 MeV with a small Pb layer (2.5 cm diameter), the beam profile and relative dose at the CAX depend on the thickness of the Pb layer (Fig. 7(a). The thicker the layer used in the calculation, the lower the relative dose at the CAX. The relative doses at the CAX changed from 20% to 5% when Pb layers with thicknesses of 2 – 8 mm were used for shielding. When a larger Pb layer (4 cm diameter) was used, the change of relative dose at the CAX was different from that of the smaller diameter layer. In Fig. 7(b), the relative dose at the CAX for the 2 mm thickness of Pb is about 13%; however, the relative dose is decreased significantly (from 5% to 3%) when the thickness of Pb is increased from 4 – 8 mm. Compared to Fig. 6, it is obvious from Fig. 7 that 2 mm of Pb is not adequate to attenuate the electron beam efficiently. However, when the thickness of the Pb layer is increased to 4 mm, the beam attenuation can be improved from 14% to 5%.

Similar effects of Pb thickness on the relative dose at the CAX are seen in Fig. 8. For 16 MeV electron beams, the relative doses at the CAX for 2 mm thickness of Pb layers with diameters of 2.5 cm (Fig. 8(a), and 4 cm (Fig. 8(b) are equal to 27% and 37%, respectively. The higher relative dose for the larger Pb layer is due to the increase of the photon contamination of the 16 MeV electron beams compared to 4 and 9 MeV.^(31)^ ^,^ ^(32)^ It is seen that 8 mm thickness of Pb cannot provide greater than 3 HVL attenuation for a Pb layer with 4 cm diameter using the 16 MeV electron beams.

### C. relative Dose at the CAX and dm

The relative dose at the CAX and $d_{m}$ reflects the attenuation of the electron beam due to the presence of the Pb layer. As discussed in Sections I V. A and IV. B above, this dose depends on the electron beam energy, size and thickness of the Pb layer. These dependencies are not significant for low electron beam energy such as 4 MeV (Fig. 9). For electron beams with energy of 9 MeV, the relative dose slightly decreases with increase of diameter of the Pb layer. This is because of a decrease of the side scatter contribution to the CAX, as the distance between the edge of the Pb layer and the CAX increases.^(33)^ ^,^ ^(34)^ In Fig. 9, it can be seen from the 9 MeV electron beams that increasing the thickness of the Pb layer from 2 to 8 mm increases the attenuation by 10% – 13%.

When the electron beam energy is increased to 16 MeV, the relative dose at the CAX is found to decrease with the diameter of the Pb layer for a thickness of 8 mm. The reason is similar to that for the 9 MeV electron beam. However, when the thickness of the Pb layer is decreased to 2 mm, it is seen that the relative dose at the CAX increases (from 26.5% to 34.5%) with an increase of the diameter of the Pb layer (from 2.5 to 4 cm) for the 16 MeV electron beams. This is because (1) the higher penetration power of the 16 MeV electron beams allows more electrons to pass through the 2 mm of Pb, and (2) such a thin Pb layer acts as a source of photon contamination and scattering medium.^(31)^ ^,^ ^(32)^

### D. Penumbra Width Varying With the Diameter of the Pb Layer

Figure 10 shows the penumbra width (20% – 80%) for different diameters of the Pb layer with 2 mm thickness and the 4, 9 and 16 MeV electron beams. It is seen that the penumbra width increases with the electron beam energy, and does not vary significantly with the diameter of the Pb layer for the 4 and 9 MeV electron beams. However, for the 16 MeV electron beams, the penumbra width decreases slightly with the diameter of the Pb layer. This is because for high electron beam energies, increased side scattering contributes more dose to the CAX.^(33)^ ^,^ ^(34)^ This increases the dose in the low‐dose region in the penumbra, resulting in a larger penumbra width. This effect is more significant for a smaller Pb layer as the edge of the shield is closer to the CAX at $d_{m}$.

### E. Shielding Area Factor

In electron radiotherapy, it is well known that a larger area of Pb compared to the size of the critical tissue should be used for shielding due to the side scatter of the electron beam. The actual size of the Pb layer, based on the size of the critical tissue at $d_{m}$, depends on the electron beam energy and dimensional characteristics of the Pb shield. A parameter called shielding area factor (Eq. 2) is introduced to predict the actual size of the Pb layer used for shielding.

Table 1 shows the shielding area factors for the 4, 9 and 16 MeV electron beams with different thicknesses and diameters of Pb. Using the 4 MeV electron beams (Table 1(a)), 5 HVL attenuation can easily be achieved. It can be seen that the shielding area factor increases with the area of the Pb shield, reflecting that a larger shielding area, compared to the shielded volume, is needed. For 9 MeV electron beams (Tables 1(b) and 1(c)), less than 5 HVL attenuation can be achieved using up to an 8 mm thickness of Pb. The attenuation is lowest for the 16 MeV electron beams, and only 3 HVL can be achieved. From Table 1, it can be seen that the efficiency of shielding is decreasing with a reduction of thickness and size of the Pb layer, in particular for the high‐energy electron beam (16 MeV). This dosimetric data is useful for radiation oncology staff in predicting the dimension of the Pb layer for shielding before the treatment planning and dose calculation.

**Table 1(d):** 16 MeV (3HVL)

	*Diameter of Pb shield (cm)*			
Thickness of Pb	2.5	3.0	3.5	4.0
6 mm	NA	0.11	0.48	0.56
8 mm	NA	0.24	0.50	0.57

## V. CONCLUSIONS

For the variation of diameter of the Pb layer, the doses at the CAX and penumbra widths at the edge of the layer and $d_{m}$ did not change significantly for the 4 MeV electron beams. When higher electron beam energies (9 and 16 MeV) were used, the dose at the CAX increased with the electron beam energy for the same thickness of Pb. For the same diameter of the Pb layer, 2 mm thickness was found to be adequate to provide shielding with 5 HVL attenuation for the 4 MeV electron beams. However, with higher electron beam energies of 9 and 16 MeV, only 4 and 3 HVL attenuation could be achieved by using 8 mm of Pb.

It was found that the beam profile at $d_{m}$ depends on: (1) the penumbra at the edge of the Pb layer; (2) the electron beam attenuation due to the Pb layer; (3) the electron side scatter contributing to the dose at the CAX; and (4) the photon contamination produced by the Pb for different electron beam energies. Points 1 through 4 are related to the size and thickness of the Pb layer and, therefore, it is concluded that the dosimetry at $d_{m}$ depends on the dimensional characteristics of the Pb layer.

The shielding area factor defined in this study was proposed to estimate the shielding effect of the Pb layer varying with its dimension. This factor is important to help the radiation oncology staff decide the size and thickness of the Pb layer prior to the computer treatment planning and dose calculation in the electron radiotherapy.

## ACKNOWLEDGEMENTS

JCLC would like to thank Dr. David Jaffray for his support in the Monte Carlo simulation at the Princess Margaret Hospital. The measurements using radiographic films were carried out in the Grand River Hospital. JCLC would also like to thank Varti Vartanian and Mitch Spiegel of Varian Medical Systems for providing detailed information of the 21 EX Linac when using electron beams, and Dr. Frank Verhaegen of McGill University for sharing his BEAMnrc input files for verification of the electron beam simulation. The assistance of Julia Publicover in proofreading this manuscript is appreciated.
